# The Nuclear Interactome of ATR7 Implicates a Chromatin-Based Repression Mechanism Controlling Oxidative Stress Tolerance and Programmed Cell Death in Arabidopsis

**DOI:** 10.3390/ijms27167445

**Published:** 2026-08-20

**Authors:** Muhammad Kamran Qureshi, Tsanko Gechev

**Affiliations:** 1 Department of Molecular Stress Physiology, Center of Plant Systems Biology and Biotechnology, 4023 Plovdiv, Bulgaria; m.k.qureshi81@gmail.com; 2 Department of Plant Breeding and Genetics, Faculty of Agricultural Sciences & Technology, Bahauddin Zakariya University, Bosan Road, Multan 60800, Pakistan; 3 Department of Molecular Biology, Plovdiv University, 4000 Plovdiv, Bulgaria

**Keywords:** abiotic stress, catalase, chromatin-associated regulation, oxidative stress, programmed cell death, superoxide dismutase

## Abstract

The redox state of the nucleus is emerging as a critical determinant of plant cell fate: reactive oxygen species (ROS) signals that originate in chloroplasts, peroxisomes, and the apoplast ultimately converge on nuclear proteins that determine whether a cell mounts a protective response or initiates programmed cell death (PCD). Loss-of-function mutations in *ATR7*, which encodes a nuclear protein specific to seed plants, confer tolerance to both paraquat- and aminotriazole-induced cell death, establishing ATR7 as a positive regulator of ROS-induced PCD. Yet how ATR7 acts at the molecular level remains unknown. In this paper, we define the ATR7 protein interactome using IP-MS of GFP-tagged ATR7 and integrate it with the *atr7* loss-of-function transcriptome to distinguish it as candidate direct molecular partners from transcriptionally regulated targets. To investigate the nuclear protein association with ATR7, we performed GFP affinity purification followed by mass spectrometry (IP-MS) using *Arabidopsis thaliana* seedlings expressing GFP-ATR7, in comparison with seedlings expressing free GFP as the negative control. The IP-MS candidates were compared with the previously published *ATR7* transcriptome data. ATR7 associates with chromatin-modifying proteins, components of the ubiquitin–proteasome system, and a broad set of stress-responsive proteins whose encoding genes are constitutively de-repressed when ATR7 is non-functional. Among the candidate proteins are those that have potential chromatin-regulatory functions, including AT1G01920 (a SET-domain protein) and HDA14, as well as components associated with ubiquitin–proteasome pathways and oxidative stress responses. Several interactors have no current functional annotation and represent candidates for novel roles in oxidative stress signalling. These findings provide the first mechanistic framework for ATR7 action and implicate nuclear chromatin-level repression as a key node in the regulation of ROS-induced PCD in plants.

## 1. Introduction

Reactive oxygen species (ROS), including superoxide radicals and hydrogen peroxide (H_2_O_2_), function both as unavoidable by-products of aerobic metabolism and as dose-dependent signalling molecules that govern plant growth, stress acclimation, and programmed cell death (PCD) [1]. The biological outcome depends upon the amount of ROS generated and the capacity of cells to perceive ROS signals. The transition from a tolerable oxidative signal to lethal oxidative damage is tightly regulated, and its disruption underlies both stress hypersensitivity and the failure to execute developmentally appropriate cell death as excessive ROS overwhelm cellular redox homeostasis, subsequently damaging cellular organelles. A key determinant of this threshold is the coordinated regulation of ROS homeostasis and transcriptional regulation of stress-protective gene networks: plants maintain repression of broad abiotic stress responses under optimal conditions, and the timely relief of this repression is critical for acclimation and survival [2]. This transcriptional reprogamming involves communication between different cellular organelles and the nucleus, engaging numerous families of ROS-induced stress-responsive transcriptional factors (TFs). These TFs regulate ROS detoxification, hormonal signalling, and PCD pathways. Transcription-factor activity is only one aspect of the ROS-dependent regulatory system. Nuclear responses to stresses are also influenced by chromatin organization, including chromatin-remodelling proteins, histone-modifying enzymes, and protein regulation, which in combination determine stress-responsive gene expression. Despite considerable progress in characterizing the TFs that activate stress-responsive genes, the nuclear proteins that impose and maintain repression of these programmes—and the molecular mechanisms through which they act—remain not completely understood.

ATR7 (AT5G21280) is a recently identified nuclear protein that functions as a positive regulator of ROS-induced PCD in *Arabidopsis thaliana*. Loss-of-function mutations in *ATR7*, generated by chemical mutagenesis, T-DNA insertion, or RNAi, confer tolerance to both paraquat, which generates superoxide radicals via photosystem I, and aminotriazole, a catalase inhibitor that causes H_2_O_2_ accumulation [3,4]. *ATR7* encodes a 302-amino acid protein with no recognisable functional domain, substantial intrinsically disordered regions, and homologs exclusively in seed plants—indicating a relatively recent evolutionary origin specific to plants [4]. Among regulatory proteins, these intrinsic disorders are common, which facilitates dynamic molecular associations with other partners. In similar analogy, the absence of a recognizable catalytic domain in ATR7 raises the possibility that its regulatory activity is associated with other proteins. However, the identification and functional significance of these candidate partners have not been investigated previously. ATR7-GFP fusion proteins localise to the nucleus in both leaf and root cells, consistent with a role in transcriptional regulation [4]. Transcriptomic analysis of the *atr7* mutant revealed that, in the absence of any exogenous stress, *atr7* plants constitutively over-express a broad suite of stress-responsive transcription factor genes—including *HSFA2, ZAT10, ZAT12,* and *DREB19*—as well as the chromatin remodeller *CHR34* and show a dramatically blunted transcriptional response when challenged with paraquat [3,4]. These observations establish ATR7 as a nuclear repressor of stress-tolerance programmes whose inactivation constitutively primes the plant against oxidative damage. However, the protein-level environment in which ATR7 operates—the molecular partners through which it exerts repression and the pathways it engages—remains entirely unknown.

To address this gap, we applied an immunoprecipitation coupled to mass spectrometry (IP-MS) approach using stable transgenic *Arabidopsis* lines expressing a GFP-ATR7 fusion protein with free GFP as the negative control and integrated the resulting dataset with the published *atr7* transcriptome to place candidate interactors in their regulatory context. The two datasets were integrated due to the fact that they provide complementary levels of information: the IP-MS identifies potential ATR7-interacting proteins, whereas transcriptomic analysis of mutants with loss of ATR7 function identifies associated gene-expression changes under oxidative stress and controlled conditions. Therefore, mapping candidate proteins identified in IP-MS analysis onto the *atr7* transcriptional response allowed us to assess whether these proteins, which are associated with ATR7, also correspond to genes exhibiting ATR7-dependent expression patterns in a converging manner across different datasets for stress-related regulatory pathways. Such harmonizations can strengthen the prioritization of candidate proteins associated with ATR7 in generating testable hypotheses in relation to molecular processes where ATR7 influences oxidative stress tolerance and ROS-mediated PCD. The data reveal associations between ATR7 and chromatin-modifying machinery, components of the ubiquitin–proteasome system, and a cluster of stress-responsive proteins whose encoding genes are constitutively de-repressed in the *atr7* mutant. Several interactors with no current functional annotation further expand the known ATR7 network. Together, these results provide the first protein-level view of the molecular environment in which ATR7 operates and offer new mechanistic insight into how nuclear repression is coupled with the control of oxidative stress tolerance and ROS-induced PCD in plants.

## 2. Results

### 2.1. Identification of Candidate ATR7-Associated Proteins by IP-MS

**Figure 1:**
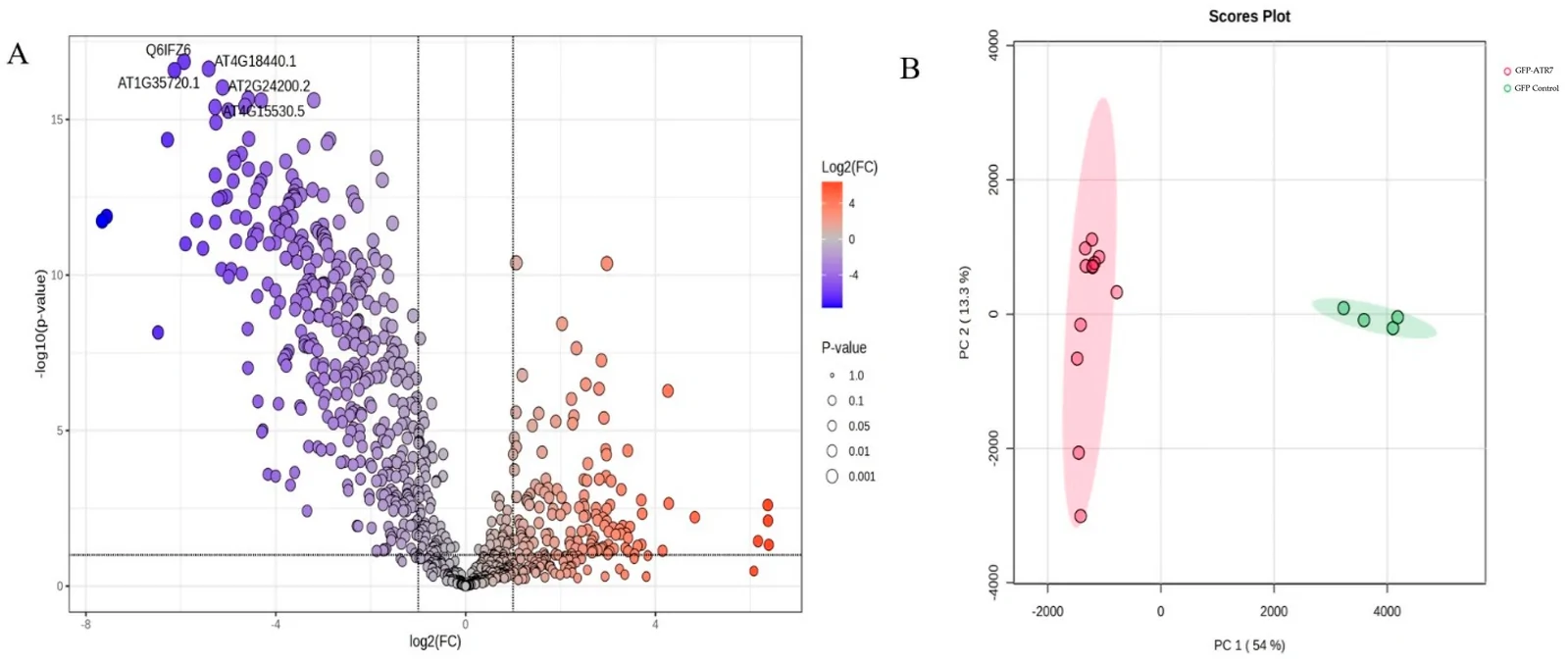
Proteins co-precipitating with ATR7 in GFP-based immunoprecipitation coupled with mass spectrometry (IP-MS). (**A**) Volcano plot displaying the differential abundance of proteins identified in GFP-ATR7 versus free-GFP control. Each point represents a single protein. The *x*-axis shows the log_2_ fold change (log_2_FC) of protein abundance in GFP-ATR7 relative to the free-GFP control; the *y*-axis shows −log_10_(*p*-value). Vertical dashed lines indicate the log_2_FC thresholds (log_2_FC > 2 and log_2_FC < −1) used to define enriched and depleted proteins, respectively. (**B**) Principal component analysis (PCA) scores plot of all IP-MS samples. Each point represents one biological replicate.

To identify proteins that co-purify with ATR7 and thereby elucidate its molecular mode of action, we performed immunoprecipitation coupled to mass spectrometry (IP-MS) using *Arabidopsis thaliana* transgenic lines (*35S::GFP-ATR7*) stably expressing a GFP-ATR7 fusion protein [4]. Replicate immunoprecipitations against free GFP-expressing plants (35S::GFP) served as the negative control for the proteins that associate with GFP, the affinity matrix, or other components during the purification process. Protein abundance in the GFP-ATR7 immunoprecipitates was compared with that of corresponding free-GFP. The representative log_2_ fold-change values for proteins enriched in the GFP-ATR7 samples are relative to the GFP control. The differential abundance was assessed by comparing the protein intensities in GFP-ATR7 immunoprecipitates with those in the control samples. The detected proteins were retained for statistical analysis. Principal component analysis of the mass spectrometry data demonstrated high reproducibility across biological replicates and clear separation of the GFP-ATR7 and GFP control samples (Figure 1B). A total of 118 proteins were enriched with a log_2_ fold change >2 and *p* < 0.05 over the GFP control (Figure 1; Supplementary Table S1), hereafter referred to as candidate ATR7-associated proteins. These encompass both potential direct binding partners, proteins recovered indirectly as components of larger complexes, and contaminants. Notably, ATR7 itself ranked among the most abundant proteins in the dataset (log_2_ FC = 19.2), confirming the efficiency and specificity of the immunoprecipitation. As is inherent to affinity purification-based approaches, the dataset is expected to include a proportion of non-specific co-precipitants. For a clearer prioritization of candidates, the proteins that meet statistical enrichment criteria were retained in the IP-MS dataset regardless of their subcellular localization or functional annotation. Accordingly, proteins well-documented as frequent contaminants in plant IP-MS experiments—including RuBisCO subunits, cytosolic GAPDH isoforms, tubulins, actins, and chaperonins—were identified among the 118 candidates and are treated with caution in the subsequent analysis. Similarly, several high-fold-enrichment hits localize strictly to the chloroplast or mitochondrion and, given the established nuclear localization of ATR7 [4], are unlikely to represent direct binding partners. Candidate interactors were therefore prioritized on the basis of subcellular localization compatibility and their absence from common affinity purification background databases.

### 2.2. The Candidate ATR7-Associated Proteome Spans Multiple Functional Categories

**Figure 2:**
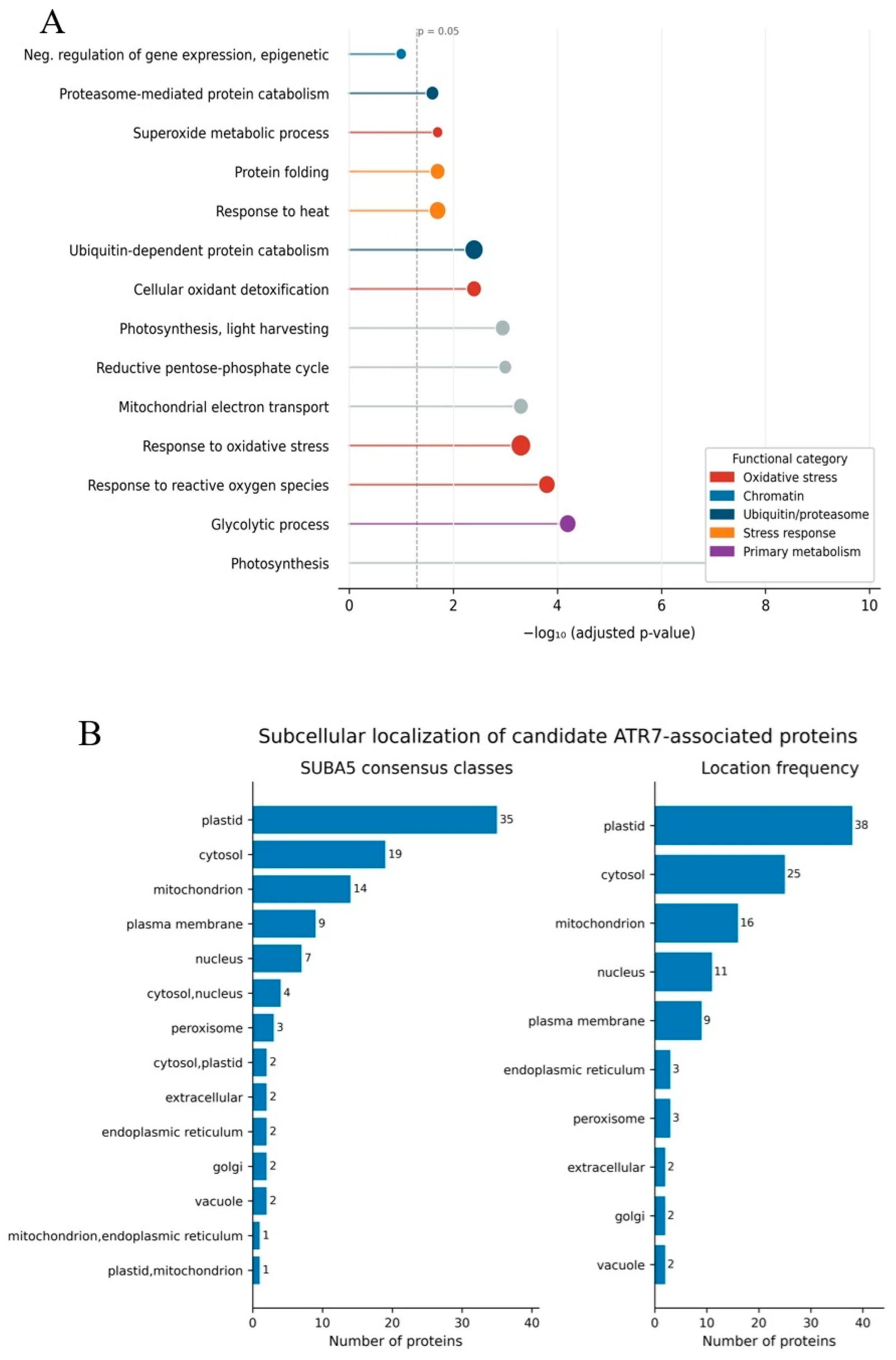
Functional annotation and subcellular localisation of candidate ATR7-associated proteins. (**A**) Gene Ontology (GO) biological process enrichment analysis of the 118 candidate ATR7-associated proteins (log_2_ fold change >2 over free-GFP control). Dot position on the *x*-axis indicates enrichment significance (−log_10_-adjusted *p*-value; Benjamini–Hochberg correction); the dashed vertical line marks the *p* = 0.05 significance threshold. Dot colour indicates the functional category assigned to each GO term. (**B**) Subcellular localisation profile of the candidate ATR7-associated proteins as assigned by the SUBA5 consensus database. Left panel shows SUBA5 consensus classes (each protein assigned to a single compartment); right panel shows location frequency counts, in which proteins with multiple predicted localisations contribute to each relevant compartment.

**Figure 3:**
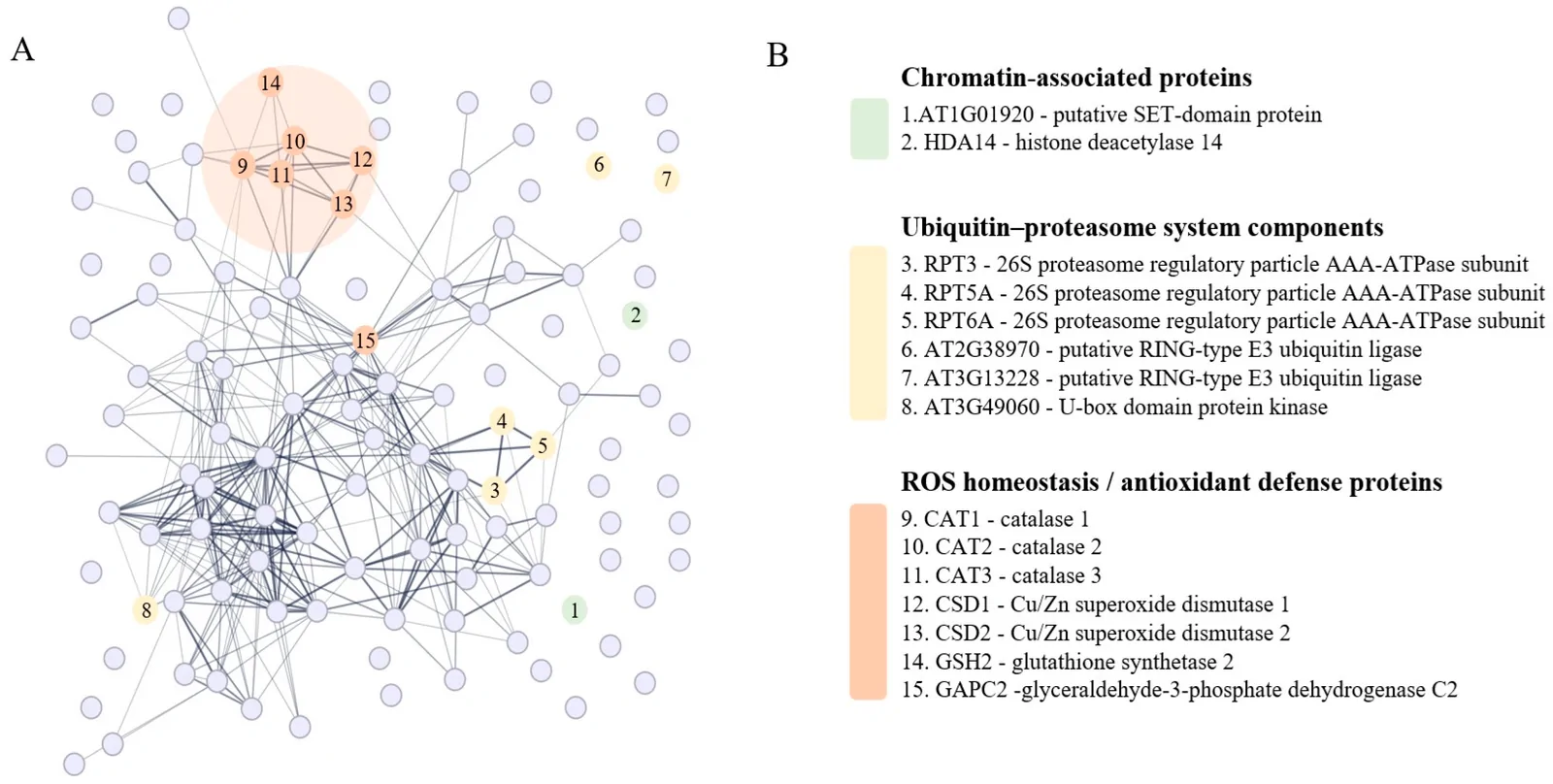
Protein–protein interaction network of candidate ATR7-associated proteins. (**A**) Network of experimentally supported and predicted protein–protein interactions among the 118 candidate ATR7-associated proteins, generated using the STRING database. Each node represents one protein; edges indicate interactions above the confidence threshold used. Fifteen proteins from three functional categories are highlighted and numbered: chromatin-associated proteins (green nodes, 1–2), ubiquitin–proteasome system components (yellow nodes, 3–8), and ROS homeostasis/antioxidant defence proteins (orange nodes, 9–15). (**B**) Key to the numbered nodes in (**A**) grouped by functional category.

Gene Ontology (GO) enrichment analysis of the 118 candidate ATR7-associated protein dataset revealed significant overrepresentation of proteins involved in oxidative stress responses and ROS scavenging, chromatin regulation, and the ubiquitin–proteasome system, as well as proteins associated with primary metabolic processes (Figure 2; Supplementary Table S1). Subcellular localisation analysis using the SUBA5 database indicated that the dataset is enriched for nuclear and cytosolic proteins, consistent with the established nuclear localisation of ATR7 [4], but also includes a substantial proportion of plastid- and mitochondrion-resident proteins, which likely reflect co-purification of abundant organellar proteins inherent to green-tissue extracts. Based upon the consensus classification, the candidate proteins were distributed across nuclear, cytosolic, mitochondrial, plastidial, and other cellular compartments (Figure 2B). Candidate interactors were therefore stratified by localization compatibility with nuclear ATR7, with plastidial and mitochondrial proteins of no documented nuclear pool treated with caution in the subsequent analysis. STRING network analysis further revealed that several functional clusters form coherent protein–protein interaction networks, with the antioxidant enzyme cluster and the proteasome regulatory particle subunits forming particularly dense nodes (Figure 3), supporting the biological relevance of these associations. In the following sections, we describe the most prominent functional groups in turn, integrating the IP-MS data with the published *atr7* transcriptome [4] to assess the biological significance of each association.

### 2.3. ATR7 Co-Purifies with Candidate Chromatin-Modifying Proteins

The highest-confidence nuclear interactors identified in the IP-MS dataset were proteins with established or predicted roles in chromatin modification. The top-ranked hit in the entire dataset was AT1G01920 (log_2_ FC = 83.7), a candidate protein carrying a SET domain, which in all characterized plant proteins mediates histone lysine methylation [5]. A bipartite nuclear localization signal predicted in AT1G01920 is consistent with the nuclear localization documented for all characterized Arabidopsis SET-domain proteins. But the potential involvement of AT1G01920 in chromatin regulation needs experimental validation. SET-domain methyltransferases deposit repressive histone marks—most commonly H3K27me3 or H3K9me2—at target loci to silence gene expression, indicating that ATR7 may be associated with AT1G01920, mechanistically coherent with its established role as a transcriptional repressor. Also recovered was HISTONE DEACETYLASE 14 (HDA14, AT4G33470; log_2_ FC = 13.0), a class II histone deacetylase with a documented nuclear pool [6]; histone deacetylases remove activating acetylation marks from histone tails, reinforcing transcriptional silencing at target loci.

Consistent with this, transcriptomic profiling of the *atr7* loss-of-function mutant [4] revealed that CHR34 (AT2G21450), a member of the CHD3/Mi-2 subfamily of ATP-dependent chromatin remodellers, is constitutively elevated more than 165-fold relative to wild type under non-stress conditions. CHD3-type remodellers in plants, exemplified by PICKLE, function as transcriptional repressors by promoting deposition of the repressive histone mark *H3K27me3* at target loci [7,8]. The constitutive upregulation of CHR34 in atr7 therefore indicates that ATR7 normally suppresses this repressive chromatin remodelling activity, suggesting that ATR7 and CHR34 may operate in a shared or partially overlapping chromatin-repression pathway whose deregulation in *atr7* contributes to the primed stress-tolerance state.

Taken together, the co-purification of a SET-domain methyltransferase and a histone deacetylase with ATR7, combined with the transcriptomic evidence that ATR7 normally suppresses CHR34—itself a repressive CHD3-type remodeller—supports a model in which ATR7 may operate as a component of a broader repressive chromatin complex that coordinately silences stress-protective gene expression under non-stress conditions.

### 2.4. Antioxidant and ROS-Homeostasis Proteins Co-Purify with ATR7

A prominent feature of the candidate ATR7-associated dataset was the enrichment of antioxidant and ROS-scavenging enzymes. Although CATALASE proteins are abundant and can appear as non-specific co-precipitants in plant IP-MS experiments, three lines of evidence suggest their recovery in this case is biologically meaningful rather than artifactual. First, all three Arabidopsis CATALASE isoforms were simultaneously recovered—CAT1 (AT1G20630, log_2_ FC = 7.86), CAT2 (AT4G35090, log_2_ FC = 2.48), and CAT3 (AT1G20620, log_2_ FC = 4.77)—and the co-recovery of all three isoforms sharing >75% sequence identity is unlikely to reflect non-specific co-precipitation. Since these proteins are highly homologous and are abundant cellular proteins, we evaluated the identification of CAT1, 2, and 3 on the basis of sequence coverage, protein intensities, unique peptides, peptide-spectrum matches, and presence of isoform-specific peptides. Second, *atr7* plants are tolerant to aminotriazole, a catalase inhibitor, providing independent genetic evidence that ATR7 normally suppresses catalase-dependent H_2_O_2_ scavenging [3]. Third, CAT2 has been shown to localise to the nucleus in addition to peroxisomes [9], making a direct nuclear encounter with ATR7 physically plausible for at least this isoform.

Additional antioxidant-related candidates included two Cu/Zn SUPEROXIDE DISMUTASE isoforms, CSD1 (AT1G08830, log_2_ FC = 2.27) and CSD2 (AT2G28190, log_2_ FC = 10.7), GLUTATHIONE SYNTHETASE 2 (GSH2, AT5G27380, log_2_ FC = 7.85), 2-Cys PEROXIREDOXIN A (AT3G11630, log_2_ FC = 2.54), and a plasma membrane AQUAPORIN (PIP2A, AT3G53420, log_2_ FC = 5.24) relevant to H_2_O_2_ flux across membranes [10,11]. The plastidial localisation of CSD2 and the plasma membrane localisation of PIP2A are incompatible with direct interaction with nuclear ATR7; unlike FeSOD isoforms, for which nuclear localisation has been reported in plants, no nuclear pool has been documented for Cu/Zn SODs. These proteins are therefore interpreted with caution, and their presence in the dataset is more likely attributable to indirect co-purification. Nevertheless, transcriptomic profiling of the *atr7* mutant [4] reveals that CSD1 and CSD2 transcripts are constitutively elevated approximately 1.8-fold and 3.2-fold, respectively, in *atr7* relative to the parental *loh2* background under non-stress conditions, and *atr7* plants show constitutively elevated glutathione levels [3], consistent with ATR7 normally repressing expression of these ROS-scavenging genes. These transcriptomic data thus provide a functional link between *ATR7* and the *Cu*/*Zn SOD* and glutathione systems at the gene expression level, even where direct protein–protein interaction remains unresolved.

GLYCERALDEHYDE-3-PHOSPHATE DEHYDROGENASE C2 (GAPC2, AT1G13440, log_2_ FC = 2.03) was also recovered. Cytosolic GAPDH isoforms are well-documented non-specific co-precipitants in affinity purification experiments; however, unlike canonical GAPDH contaminants, GAPC2 undergoes H_2_O_2_-induced nuclear translocation in Arabidopsis, where it interacts with transcription factors to modulate stress gene expression [12]. Given that ATR7 itself is induced by H_2_O_2_ [4], its recovery may reflect a genuine stress-state nuclear interaction rather than non-specific co-precipitation.

### 2.5. ATR7 Associates with Components of the Ubiquitin–Proteasome System

Three subunits of the 19S regulatory particle of the 26S proteasome were identified as candidate ATR7-associated proteins: RPT3 (AT5G58290, log_2_ FC = 2.91), RPT5A (AT3G05530, log_2_ FC = 7.53), and RPT6A (AT5G19990, log_2_ FC = 5.46). The co-recovery of three AAA-ATPase subunits of the same complex substantially reduces the probability that any individual hit reflects non-specific co-precipitation. Complementing this, two RING-type E3 ubiquitin ligases (AT2G38970, log_2_ FC = 10.3; AT3G13228, log_2_ FC = 8.61) and a U-box domain protein kinase (AT3G49060, log_2_ FC = 4.69) were also recovered; RING-type and U-box E3 ligases catalyse the transfer of ubiquitin to substrate proteins, targeting them for proteasomal degradation. At the transcriptomic level, ubiquitin-dependent protein degradation pathways are among the most significantly upregulated biological processes in *atr7* [4], providing independent support for a functional connection between ATR7 and the ubiquitin–proteasome system (UPS). Interestingly, the co-recovery of both proteasome subunits and E3 ligases alongside the SET-domain methyltransferase identified in Section 3.2 is mechanistically coherent, as histone methylation marks deposited by SET-domain proteins are known to recruit E3 ubiquitin ligases, and ubiquitination in turn regulates chromatin remodeller activity—suggesting that the chromatin and UPS components of the ATR7-associated proteome may form part of an interconnected regulatory axis. Because ATR7 is an intrinsically disordered protein with no recognisable functional domain (Sujeeth et al., 2020) [4], a plausible interpretation is that the proteasome subunits and E3 ligases reflect active ubiquitin-mediated turnover of ATR7 itself, rather than ATR7 directly regulating proteasome function. Intrinsically disordered nuclear proteins with low-complexity sequences are well-established substrates of the UPS, and regulated proteolysis represents a common mechanism for controlling the abundance of nuclear scaffold proteins.

At the transcriptomic level, GO enrichment analysis of genes upregulated in *atr7* reveals significant overrepresentation of ubiquitin-dependent protein degradation and proteasome-related terms [4], consistent with a functional connection between *ATR7* and the *UPS* at the gene expression level. This pathway-level upregulation in atr7 is notable in that it could reflect two non-mutually exclusive scenarios: ATR7 may normally repress components of the UPS as part of its broader transcriptional repressor activity, or alternatively, the upregulation of degradation machinery in atr7 may represent a compensatory response to the loss of ATR7-mediated chromatin repression, serving to clear de-repressed or misfolded proteins. Precedent for UPS involvement in ROS-induced PCD in plants already exists—several proteasome subunit genes and ubiquitin-related genes were found to be strongly induced during H_2_O_2_-triggered cell death [13]—placing the ATR7–UPS connection in a broader biological context.

### 2.6. Integration of IP-MS Data with the atr7 Transcriptome Reveals a Coherent Repressor Network

To place the candidate ATR7-associated proteins in the broader context of ATR7 function, we integrated the IP-MS dataset with the published RNA-seq transcriptome of the *atr7* loss-of-function mutant [4]. Under non-stress conditions, 620 genes are constitutively upregulated and 434 downregulated in *atr7* relative to the parental *loh2* background, with 32% of the most strongly upregulated genes encoding predicted nuclear proteins—consistent with ATR7 functioning primarily as a nuclear regulator. Among the most strongly de-repressed genes are the abiotic stress-responsive transcription factors *HSFA2*, *ZAT10*, *ZAT12*, and *DREB19*, each constitutively elevated several-fold in *atr7* under non-stress conditions [4]. Importantly, none of these transcription factors were recovered as ATR7-associated proteins in the IP-MS dataset—they represent transcriptional targets of the ATR7 repressor network rather than direct physical interactors. Strikingly, paraquat treatment induces ≥2-fold expression changes in 7623 genes in wild-type loh2, whereas only 1942 genes respond comparably in *atr7*, indicating that a large portion of the acute ROS-transcriptome is already constitutively pre-activated in the absence of ATR7 [4].

**Figure 4:**
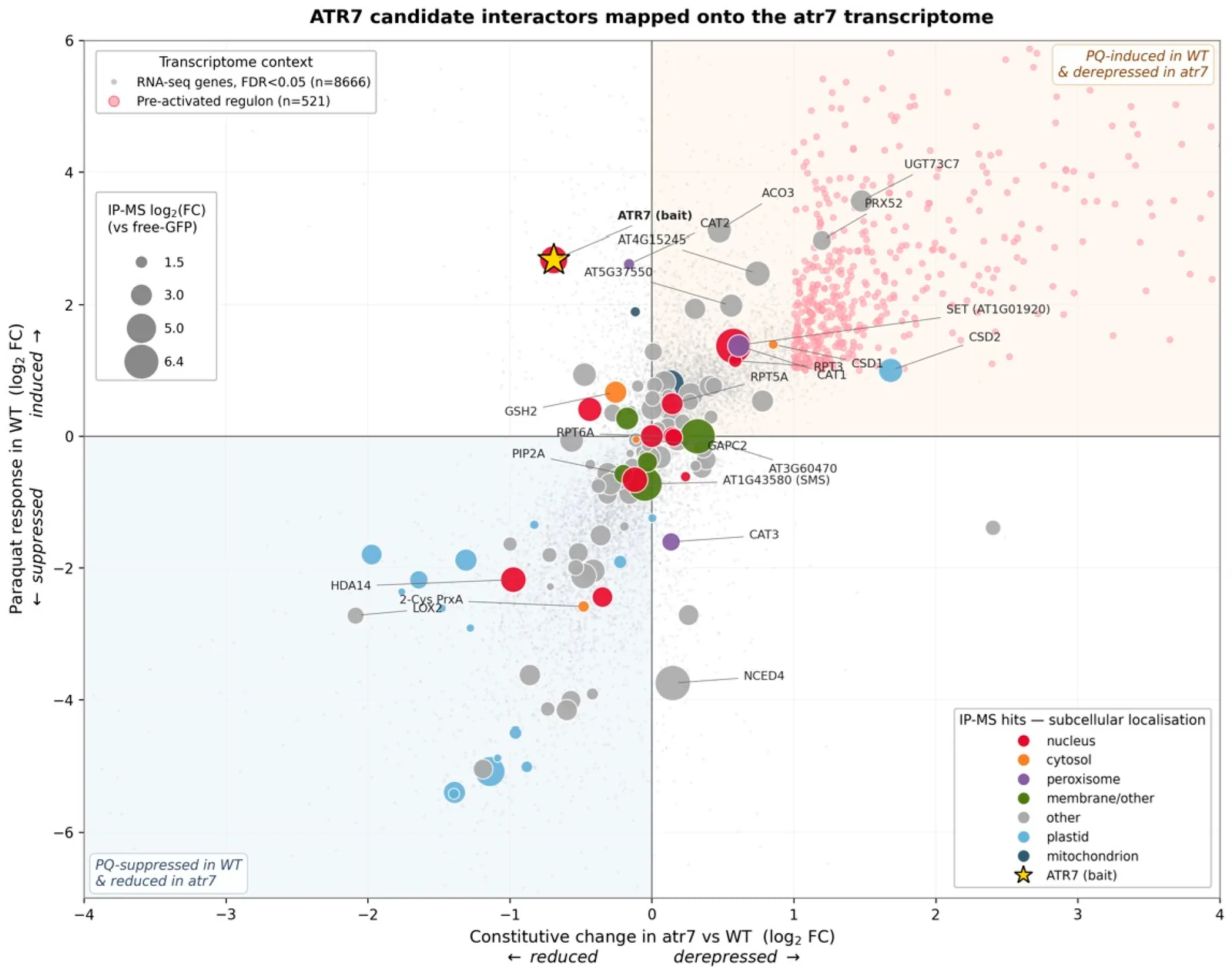
ATR7 candidate protein interactors plotted on the atr7 transcriptomic response under paraquat treatment. The position of candidates within the transcriptomic landscape shows concordance between ATR7-dependent gene-expression patterns and protein co-purification. The horizontal axis shows mRNA change in atr7 vs. loh2 under control conditions (log_2_ FC; Sujeeth et al., 2020 [4]); the vertical axis shows the paraquat response in WT (loh2-PQ vs. loh2-MS, log_2_ FC). Background points are from a two-way ANOVA (genotype × treatment) on the same dataset: small grey dots (*n* = 8666) mark genes with a significant genotype-by-treatment interaction (FDR < 0.05); pink dots (*n* = 521) are the “pre-activated” set—genes constitutively elevated in atr7 (log_2_ FC > 1), induced by paraquat in WT (log_2_ FC > 1), and showing a blunted paraquat response in atr7 (negative interaction term, FDR < 0.05). Circle colour indicates predicted subcellular localisation; circle size reflects IP-MS enrichment over free-GFP control (log_2_ FC, scale shown).

To examine where the IP-MS hits sit within this transcriptome, we re-analysed the four-condition RNA-seq data using a two-way ANOVA (genotype × treatment)—a complementary approach to the fold-change comparisons cited above. This identified 8666 genes whose paraquat response depends significantly on ATR7 (FDR < 0.05; Figure 4). The horizontal axis shows the change in mRNA level in *atr7* compared to *loh2* under control conditions; so, genes to the right of zero are present at higher levels in *atr7*—the pattern expected if ATR7 normally represses them. The vertical axis shows how strongly the gene responds to paraquat in WT plants. The 8666 genes that pass this significance threshold (FDR < 0.05) are shown as small grey dots, illustrating the overall shape of the atr7 transcriptome. Among these, a stricter 521-gene “pre-activated” set—genes already elevated in *atr7* under control conditions, induced by paraquat in WT, and showing a weaker paraquat response in atr7 than in WT (significant negative interaction term, FDR < 0.05)—is highlighted in pink. This pre-activated set captures the stress-protective regulon that is constitutively switched on when ATR7 is missing. Most of the strongest candidates for ATR7-mediated repression—including the SET-domain protein AT1G01920, CAT1 and CAT2, CSD1, CSD2, RPT3, AT4G15245, AT5G37550, UGT73C7, and PRX52—fall into the upper-right quadrant, sitting next to or within the pre-activated regulon, with UGT73C7 and PRX52 in particular passing all of the strict criteria for membership. The lower-left quadrant, by contrast, is dominated by photosynthesis and plastid-encoded proteins whose transcripts are suppressed by paraquat and reduced in *atr7*—a pattern shared with much of the rest of the transcriptome and consistent with the general shutdown of photosynthesis under oxidative stress. Combined with the incompatibility of these proteins with the nuclear localisation of ATR7, this strongly supports their interpretation as co-purifying contaminants rather than direct partners.

**Figure 5:**
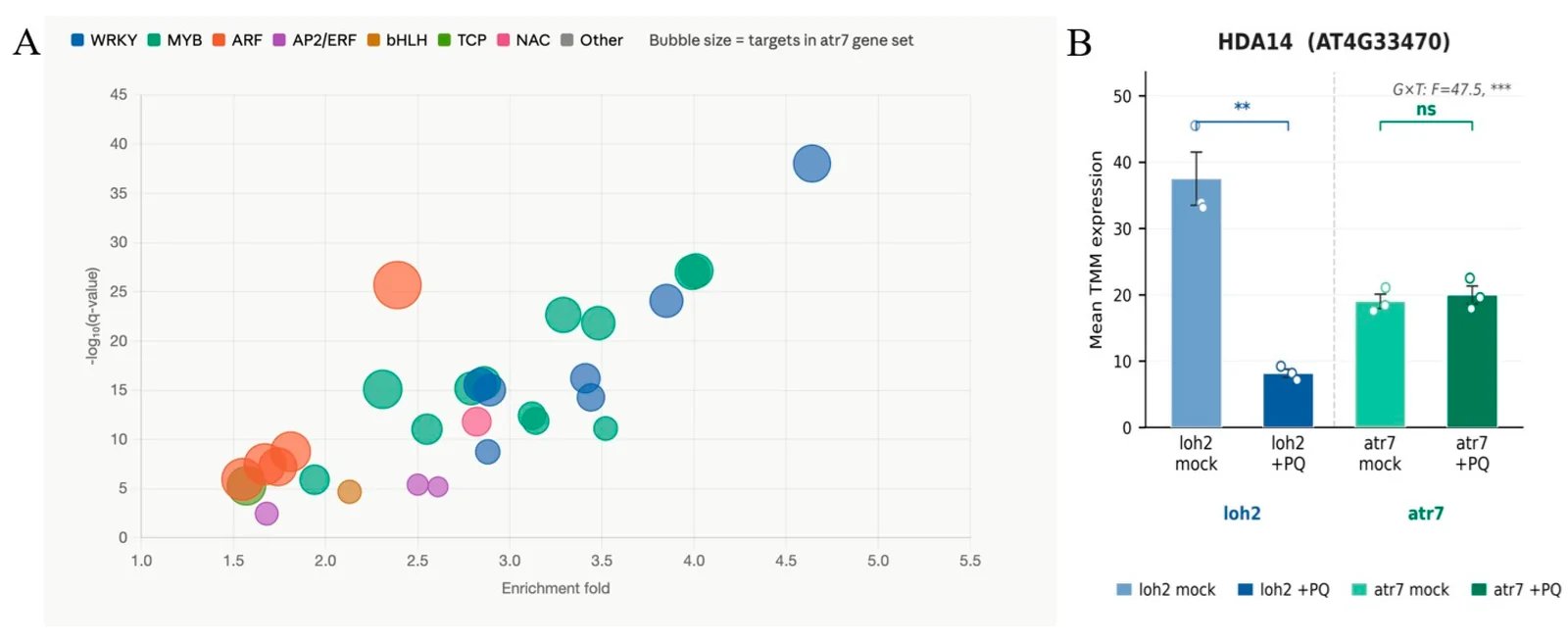
Transcription factor binding site enrichment in the *ATR7* regulon and transcriptional regulation of HDA14. (**A**) Bubble plot of transcription factor (TF) binding site enrichment analysis in the set of genes differentially expressed in *atr7* relative to the parental *loh2* background. The *x*-axis shows enrichment fold, and the *y*-axis shows statistical significance (−log_10_ q-value). Bubble size is proportional to the number of target genes in the *atr7* gene set regulated by each TF. Bubbles are colour-coded by TF family as indicated in the legend. (**B**) Mean TMM-normalised expression of *HDA14 (AT4G33470*; *Histone Deacetylase 14*) in loh2 and atr7 plants under mock and paraquat (+PQ) treatment conditions. Bars show mean ± SEM (*n* = 3 biological replicates per condition; individual data points shown). The genotype × treatment interaction term from a two-way ANOVA is indicated above the plot (G × T: F = 47.5, *** *p* < 0.001). Brackets indicate pairwise comparisons: ** *p* < 0.01; ns, not significant.

To determine which transcription factor families are predicted to regulate the ATR7-repressed gene set and thereby place the chromatin complex identified by IP-MS in its broader regulatory context, we submitted the constitutively upregulated genes in *atr7* to TF enrichment analysis using PlantRegMap/PlantTFDB 5.0. A total of 136 TFs were significantly enriched (*q* < 0.05), dominated by the WRKY and MYB families, with WRKY40 as the top-ranked regulator (Figure 5). Although direct interactions between the specific IP-MS proteins identified in this paper and the enriched WRKY/MYB TFs have not been experimentally demonstrated, published evidence establishes that SET-domain histone methyltransferases regulate *WRKY40* expression at the chromatin level [14]; that class I/II HDACs directly repress WRKY38, WRKY62, and WRKY53 loci [15]; and that 26S proteasome RPT subunits mediate WRKY TF turnover [16] and influence histone dynamics [17]. The co-purification of functionally equivalent enzyme classes with ATR7 therefore suggests that the ATR7 complex may regulate WRKY-family TF activity at multiple levels—chromatin repression of *WRKY* loci and proteasome-mediated degradation of activated WRKY proteins. The co-recovery of two RING-type E3 ligases alongside HDA14 and the SET-domain methyltransferase AT1G01920 further raises the possibility that the ATR7 complex coordinates histone methylation, deacetylation, and ubiquitination in a concerted repressive cascade [18]—a degree of chromatin regulatory integration well described at Polycomb-repressed loci in animals but poorly characterized in plants.

To probe whether chromatin-modifying partners identified by IP-MS show ATR7-dependent regulation under oxidative stress, we examined the expression of *HDA14* across all four RNA-seq conditions (Figure 5B). HDA14 (AT4G33470), the class II histone deacetylase recovered in the ATR7 IP-MS (log_2_FC = 13.0), was strongly repressed by paraquat in the *loh2* parental background (−2.2 log_2_FC; *p* < 0.01) but showed no significant change in *atr7* under the same treatment (Figure 5B). Two-way ANOVA confirmed a highly significant genotype × treatment interaction (F = 47.5, *p* = 1.3 × 10^−4^), indicating that PQ-induced repression of HDA14 is ATR7-dependent. These data support a functional link between ATR7 and chromatin-associated repression that is dynamically modulated under oxidative stress.

## 3. Discussion

### 3.1. ATR7 as a Candidate Intrinsically Disordered Scaffold Component Associated with Chromatin Regulatory Complex

A central and previously unresolved question about ATR7 has been how a protein with no recognizable enzymatic domain, no Pfam-annotated motifs, and significant intrinsically disordered regions can function as a transcriptional repressor [4]. The IP-MS data presented in this paper offer a possible mechanistically coherent answer: rather than acting enzymatically, ATR7 likely functions as a nuclear scaffold or adaptor protein that recruits chromatin-modifying machinery to target loci, as predicted in IP-MS data. The co-purification of a candidate SET-domain histone methyltransferase (AT1G01920) and a class II histone deacetylase (HDA14) with ATR7 suggests that ATR7 may nucleate or stabilize a repressive chromatin complex capable of depositing repressive histone marks and removing activating acetylation at stress-responsive gene promoters. A testable model is that ATR7 acts as an adaptor or a scaffold within the predicted regulatory assemblies. This mode of action—a disordered scaffold recruiting enzymatically active chromatin modifiers—is well precedented in eukaryotic biology. Intrinsically disordered proteins frequently function as hubs in chromatin regulatory complexes precisely because their conformational flexibility allows simultaneous engagement with multiple structured partners, and many established transcriptional repressors in both plants and animals lack intrinsic enzymatic activity yet recruit histone-modifying machinery through disordered interaction surfaces [19,20].

The evolutionary context of ATR7 reinforces this interpretation. ATR7 is specific to seed plants, with no homologs in algae, mosses, ferns, fungi, or animals [4], indicating it represents a recently evolved regulatory layer superimposed on a conserved chromatin machinery. The SET-domain methyltransferase and histone deacetylase families it appears to recruit are deeply conserved across eukaryotes—it is ATR7 itself, not its partners, that is evolutionarily novel. This pattern is consistent with a model in which a newly evolved, lineage-specific disordered protein has been co-opted to direct pre-existing conserved chromatin-modifying enzymes to a novel set of target genes, in this case, genes governing oxidative stress tolerance and ROS-induced PCD. Analogous examples of lineage-specific intrinsically disordered proteins repurposing conserved chromatin machinery for novel regulatory functions have been described in yeast and mammals [21,22], but to our knowledge, ATR7 represents the first such candidate in the context of plant stress-induced PCD.

The interconnection between the chromatin and ubiquitin–proteasome components of the ATR7-associated proteome adds a further layer of mechanistic interest. Histone H3 methylation marks deposited by SET-domain proteins serve not only as direct repressive signals but also as docking sites for E3 ubiquitin ligases that ubiquitinate adjacent histones, creating a coupled methylation–ubiquitination repressive cascade [18]. The co-recovery of two RING-type E3 ligases alongside AT1G01920 and HDA14 in the ATR7 IP-MS is therefore mechanistically coherent and raises the possibility that the ATR7-associated complex coordinates histone methylation, deacetylation, and ubiquitination in a concerted repressive programme—a degree of chromatin regulatory integration that has been well described at Polycomb-repressed loci in animals but remains poorly characterized in plants. Nevertheless, BiFC or split-luciferase assays, reciprocal co-immunoprecipitation along with chromatin-based approaches like CUT&RUN or ChIP are necessary to determine the functional role of ATR7-interacting proteins.

### 3.2. A Proposed Model Linking ATR7-Mediated Chromatin-Mediated Repression of Antioxidant Genes as a Mechanism for ROS Amplification During PCD

A recurring feature of plant ROS-induced PCD is the biphasic oxidative burst, in which an initial moderate elevation of ROS is followed by a second, substantially stronger peak that commits the cell to death [23,24]. The molecular basis of this second burst has been attributed partly to active ROS generation and partly to suppression of ROS-scavenging capacity, but the mechanisms underlying the latter have remained poorly defined. The data presented in this paper offer a possible candidate molecular explanation: ATR7-mediated chromatin repression of antioxidant gene expression may constitute a transcriptional switch that suppresses scavenging capacity precisely when ROS levels begin to rise, amplifying the oxidative signal toward the threshold required for PCD commitment.

The key insight from our data is that the connection between ATR7 and antioxidant enzymes operates at the level of gene expression rather than direct enzyme inhibition. The co-purification of all three CATALASE isoforms with ATR7 is most parsimoniously explained by their capture at chromatin during ATR7-mediated transcriptional repression of CAT genes—consistent with the nuclear localization of CAT2 demonstrated by Al-Hajaya et al. (2022) [9] and with the tolerance of *atr7* plants to aminotriazole, which becomes meaningful only if ATR7 normally limits catalase abundance or activity at the transcriptional level [3]. This reframing has an important implication: it places the ATR7–antioxidant axis firmly in the nucleus rather than the cytosol or peroxisome, resolving what would otherwise be a paradox between ATR7’s nuclear localisation and the predominantly peroxisomal residence of catalases.

The superoxide dismutase data add a further regulatory dimension. The co-recovery of CSD1 and CSD2 alongside the enrichment of miR398 family members under paraquat stress suggests that ATR7 may contribute to a coordinated multi-level suppression of superoxide scavenging—operating through chromatin repression of *CSD* gene transcription at the nuclear level through an unknown mechanism while miR398-mediated post-transcriptional silencing provides an additional layer of control in the cytosol. This kind of transcriptional and post-transcriptional co-regulation of the same targets by overlapping regulatory networks is well established for stress-responsive genes in plants [25] but has not previously been connected to a chromatin-based repressor. The present data suggest ATR7 may act as a chromatin-level coordinator of this multi-tier silencing programme.

It is worth considering why a plant would evolve a dedicated repressor of its own antioxidant system. One perspective is that ROS-induced PCD serves an important adaptive function—eliminating pathogen-infected or irreparably damaged cells—and that premature or excessive antioxidant scavenging would abort this response. From this viewpoint, ATR7 functions not as a promoter of cell death per se but as a guardian of the PCD threshold, ensuring that the oxidative signal is sufficiently amplified before an irreversible commitment to death is made. The specificity of ATR7 for oxidative and osmotic stresses (but not heat or cold [4]) is consistent with this interpretation—it is precisely the stresses that generate ROS as a primary signal where tight regulation of scavenging capacity would be most consequential for the binary stress-tolerance versus PCD decision.

### 3.3. Regulated Proteolysis as a Potential Mechanism for Controlling ATR7 Abundance

The co-purification of three 26S proteasome regulatory particle subunits and three E3 ubiquitin ligases with ATR7 raises the question of what functional relationship exists between ATR7 and the ubiquitin–proteasome system. Two interpretations are possible: ATR7 could regulate UPS activity as part of its broader repressor function, or ATR7 could itself be a substrate of the UPS, with the co-purified components representing the degradation machinery acting on ATR7. We favour the latter interpretation for several reasons.

First, ATR7 carries the molecular hallmarks of a UPS substrate. Intrinsically disordered proteins with low-complexity sequences are among the most efficiently degraded substrates of the 26S proteasome, and disordered regions frequently serve as degradation signals (degrons) recognised by E3 ubiquitin ligases [26]. ATR7’s lack of any recognizable structured domain, combined with its significant predicted disorder [4], places it squarely in the category of proteins that are subject to constitutive or condition-dependent proteasomal turnover. Second, ATR7 is an angiosperm-specific protein with no homologs in organisms where the core 26S proteasome subunits are deeply conserved—it would be evolutionarily parsimonious for a recently-evolved disordered protein to be regulated by, rather than to regulate, an ancient, conserved complex.

Third, and most importantly, regulated proteolysis of a transcriptional repressor provides an elegant mechanistic explanation for how stress-induced de-repression could be achieved rapidly and reversibly. If ATR7 protein abundance is controlled by UPS-mediated degradation, then conditions that trigger its destruction—such as post-translational modifications induced by oxidative stress—would lead to rapid de-repression of antioxidant and stress-protective genes without requiring changes in *ATR7* mRNA levels. This would be consistent with the known induction of ATR7 transcription by H_2_O_2_ [4]: stress simultaneously induces ATR7 synthesis and, potentially, its degradation, creating a dynamic balance that could fine-tune the amplitude and duration of the repressive response. Precedent for this kind of transcriptional repressor whose activity is regulated by UPS-mediated proteolysis rather than by post-translational modification of activity per se is well established in plant signalling—the JAZ repressors of jasmonate signalling and the DELLA repressors of gibberellin signalling both follow exactly this logic [27,28]— though neither involves an intrinsically disordered scaffold of the kind proposed in this paper for ATR7.

The transcriptomic upregulation of ubiquitin-dependent degradation pathways in *atr7* [4] is also consistent with this model, though it is not by itself discriminating: the upregulation could reflect compensatory induction of the UPS in the absence of *ATR7* repression, or it could reflect the fact that *ATR7*’s gene targets include UPS components. Resolving this will require direct measurement of ATR7 protein stability—for example, by cycloheximide chase assays in wild-type and proteasome-inhibited plants—which represents an important priority for future work.

## 4. Materials and Methods

### 4.1. Plant Material

*Arabidopsis thaliana* ecotype Columbia-0 overexpressing ATR7-GFP fusion protein (35S::ATR7-GFP) and free GFP-expressing plants (35S::GFP) as a control (Sujeeth et al. 2020) [4] were grown on Murashige and Skoog plant growth media under standard growth conditions (210C, 120 µE light intensity, 16/8 day/night photoperiod). Paraquat (1 µM) was added to the plant growth media to induce ATR7 gene expression. Two-week-old seedlings were used as plant material for the subsequent proteome Immunoprecipitation–Mass Spectrometry (IP–MS) analysis.

### 4.2. Immunoprecipitation–Mass Spectrometry Analysis

Three grams of ATR7-GFP seedlings were harvested in liquid nitrogen, with three biological replicates. The ATR7-GFP fusion protein was isolated using iST GFP-Trap Kit (Article no. PO00049) by Preomics chromotek, Billerica, MA, USA. The samples were isolated according to the protocol provided by the manufacturer. Immunoprecipitations against free GFP-expressing plants (35S::GFP) served as the negative control. The resulting peptides were analysed on Orbitrap Fusion™ Lumos™ Tribrid™ Mass Spectrometer, Thermo Fisher Scientific, Waltham, MA, USA, using nanoflow C18 reverse-phase liquid chromatography using a 15 cm column (Zorbax, Agilent, Santa Clara, CA, USA) and a Dionex Ultimate 3000 RSLC nano-UPLC system (Thermo, Waltham, MA, USA). Peptides were eluted with up to a 120 min, 4% to 40% acetonitrile gradient. Spectra were acquired using the default settings for peptide identification, employing HCD activation, resolution 60,000 (MS) and 15,000 (MS2), and 60 s dynamic exclusion.

### 4.3. Protein Quantification and Statistical Analysis

The measured spectra were recalibrated and searched against the reference protein database by Proteome Discoverer 2.4, employing Sequest HT (Thermo Fisher Scientific) and MS Amanda 2.0 with the following parameters: Enzyme—trypsin, max two missed cleavage sites; modifications—carbamidomethyl (Cys) and up to three dynamic modifications, including met oxidation, Asn/Gln deamidation, and N-terminal acetylation; MS1 tolerance—5 ppm (MS Amanda), 10 ppm (Sequest); MS2 tolerance—0.02 Da (MS Amanda), 0.1 Da (Sequest). The quantitative differences were determined by Minora, employing precursor ion quantification followed by normalization and background-based *t*-test. Each sample was analysed by two injections (1 µL and 2 µL) as technical replicates. log_2_-normalised abundances were averaged across the two injections of each sample prior to statistical testing, so that all tests were performed at the biological-replicate level (*n* = 6 ATR7-GFP versus free-GFP).

Protein abundance values were normalized and subsequently subjected to statistical analysis. Differential protein abundance between GFP-ATR7 and free-GFP control immunoprecipitates was assessed using *t*-test. Proteins with log_2_FC > 2 and *q* < 0.05 were considered significantly enriched in GFP-ATR7 immunoprecipitates.

### 4.4. Gene Ontology and Functional Classification Analysis

Gene Ontology (GO) biological-process enrichment analysis was performed using g:Profiler (g:GOSt; version e114_eg62_p19_27110d83) against Arabidopsis thaliana annotations. The input comprised the 118 candidate ATR7-associated proteins, submitted as AGI locus identifiers. No filtering for abundant organellar proteins or literature-flagged background proteins was applied before enrichment; so, the analysis reflects the composition of the unfiltered candidate list. Statistical significance was assessed against the annotated-genome background (g:Profiler “annotated” domain scope) using the g:SCS multiple-testing procedure, which accounts for the hierarchical dependency between GO terms, at a threshold of 0.05. For presentation in Figure 2A, enriched terms were grouped manually into five descriptive functional categories; this grouping is illustrative and was not derived algorithmically.

Subcellular localisation was assigned using SUBA5 (https://suba.live), which integrates experimental proteomic, fluorescent-protein, and protein–protein interaction data with sequence-based predictions. Each protein was assigned a single consensus compartment by the SUBAcon classifier; these assignments are shown in the left panel of Figure 2B. Because many proteins carry evidence for more than one compartment, the right panel of Figure 2B additionally reports location-frequency counts, in which a protein with multiple documented localisations contributes to each relevant compartment. Proteins recurrently reported as background in plant affinity-purification experiments—including RuBisCO subunits, cytosolic GAPDH isoforms, tubulins, actins, and chaperonins—were annotated as such on literature grounds.

Protein–protein association networks among the 118 candidate ATR7-associated proteins were constructed using STRING v12.0 with Arabidopsis thaliana (NCBI tax id 3702) as the reference organism. AGI locus identifiers were submitted as a multiple-protein query and mapped to STRING identifiers. The full STRING network was used, in which edges denote functional associations of any kind, with all active interaction sources enabled (textmining, experiments, curated databases, co-expression, neighbourhood, gene fusion, and co-occurrence) and a minimum required interaction score of 0.400 (medium confidence). Disconnected nodes were retained in the displayed network. Edge thickness in Figure 3A is proportional to the STRING combined score. Nodes were coloured manually to indicate the three functional categories highlighted in the figure; this annotation was applied for presentation and is not derived from STRING clustering.

### 4.5. Reanalysis of the atr7 RNA-Seq Dataset

The RNA-seq dataset of Sujeeth et al. (2020) [4] (NCBI BioProject PRJNA475098) comprises four conditions—loh2 control (MS), loh2 paraquat (PQ), atr7 MS, and atr7 PQ—with three biological replicates each. Raw reads were not reprocessed; the analysis used the TMM-normalised expression values reported in that study. So, read processing, reference genome, and annotation versions are as described therein.

All analyses were performed in R (version 4.4.1) within RStudio (version 2024.04). TMM values for all 27,655 genes were log_2_-transformed and fitted gene-by-gene by two-way ANOVA using the aov function of the stats package, with genotype, treatment, and their interaction as fixed effects. The interaction term, (atr7 PQ−atr7 MS)−(loh2 PQ−loh2 MS), is negative when the paraquat response is blunted in atr7. Interaction *p*-values were adjusted across all tested genes by the Benjamini–Hochberg procedure (p.adjust, method = “BH”), and 8666 genes showed a significant genotype × treatment interaction at FDR < 0.05. A pre-activated subset of 521 genes was defined by four simultaneous criteria: FDR < 0.05, a negative interaction term, constitutive elevation in atr7 (log_2_FC > 1), and paraquat induction in loh2 (log_2_FC > 1). Contrasts, F statistics, and raw and adjusted *p*-values for the complete gene set are provided in Table S2.

Because the model was applied to previously normalised values rather than raw counts, it does not estimate count-level dispersion, and the primary differential-expression calls remain those of the original study. The ANOVA is used in this paper to stratify that dataset according to ATR7 dependence rather than to redefine the set of differentially expressed genes.

Expression of HDA14 (AT4G33470) was tested across the same four conditions using the identical model (*n* = 3 per condition), followed by Tukey’s honestly significant difference test (TukeyHSD) for pairwise comparisons between conditions.

### 4.6. Transcription-Factor Target Enrichment Analysis

Transcription-factor (TF) target enrichment was performed using the TF Enrichment tool of PlantRegMap (https://plantregmap.gao-lab.org/tf_enrichment.php, accessed on 14 August 2026), which queries the regulatory interactions compiled in PlantTFDB 5.0, with *Arabidopsis thaliana* (TAIR10) as the reference species. The input gene set comprised the 620 genes constitutively upregulated in *atr7* relative to the parental *loh2* background under non-stress conditions, submitted as AGI locus identifiers. Regulatory interactions in PlantRegMap are curated from the published literature and ChIP-seq experiments or inferred by combining experimentally determined TF binding motifs with regulatory-element data; all available evidence types (motif, motif_DGF, motif_CE, and FunTFBS) were enabled. Over-representation of the predicted targets of each TF within the input set was assessed by Fisher’s exact test, using the set of genes carrying regulatory annotations in PlantRegMap as the background.

## 5. Conclusions

How a nuclear protein with no enzymatic domain and no homologs outside seed plants can function as a critical regulator of ROS-induced programmed cell death has been an open question since ATR7 was first characterised genetically. The IP-MS interactome reported in this paper provides the first protein-level understanding. ATR7 co-purifies with a SET-domain histone methyltransferase and a class II histone deacetylase, supporting a model in which ATR7 may function as an intrinsically disordered nuclear scaffold that recruits repressive chromatin-modifying machinery to stress-protective gene loci. This proposed model may account for the constitutive stress-primed transcriptome, the aminotriazole tolerance, and the resistance to ROS-induced PCD observed in *atr7* loss-of-function plants. The co-purification of 26S proteasome subunits and E3 ubiquitin ligases further suggest that ATR7 abundance is subject to regulated proteolysis, raising the possibility that stress-induced modulation of ATR7 stability may contribute as a molecular switch between repression and de-repression of the antioxidant programme.

Integration with the *atr7* transcriptome reinforces this model: the most strongly de-repressed transcripts encode precisely the stress-protective functions represented by the co-purified proteins. The enrichment of miR398 family members alongside CSD1 and CSD2 further suggests that suppression of superoxide scavenging may involve at both transcriptional and post-transcriptional levels—a degree of regulatory integration not previously associated with a chromatin scaffold in the context of plant PCD.

Although this study presents several potential ATR7-interacting proteins and outlines an emerging model of ATR7 action, it has limitations. More experiments are needed to demonstrate direct binding and recruitment to chromatin, enzymatic activity, or repression of specific loci. Furthermore, genetic studies (e.g., knockout mutants or/and genome edited plants) are needed to confirm the involvement of the implicated proteins in the ATR7 mode of action.

ATR7 represents a recently evolved, seed-plant-specific node that may couple with nuclear chromatin state to the control of oxidative stress tolerance and ROS-induced cell death, adding a new regulatory layer to the plant stress-response network.

## Data Availability

The original contributions presented in this study are included in the article/Supplementary Materials. Further inquiries can be directed to the corresponding author.

## Supplementary Materials

The following supporting information can be downloaded at https://www.mdpi.com/article/10.3390/ijms27167445/s1.
